# Functional Coordination among the Golgi Complex, the Centrosome and the Microtubule Cytoskeleton during the Cell Cycle

**DOI:** 10.3390/cells11030354

**Published:** 2022-01-21

**Authors:** Fabiola Mascanzoni, Roberta Iannitti, Antonino Colanzi

**Affiliations:** Institute of Experimental Endocrinology and Oncology “G. Salvatore” (IEOS), National Research Council (CNR), Via P. Castellino 111, 80131 Naples, Italy; f.mascanzoni@ieos.cnr.it (F.M.); r.iannitti@ieos.cnr.it (R.I.)

**Keywords:** Golgi complex, centrosome, microtubules, cell migration, mitosis, mitotic spindle

## Abstract

The Golgi complex of mammalian cells is organized in a ribbon-like structure often closely associated with the centrosome during interphase. Conversely, the Golgi complex assumes a fragmented and dispersed configuration away from the centrosome during mitosis. The structure of the Golgi complex and the relative position to the centrosome are dynamically regulated by microtubules. Many pieces of evidence reveal that this microtubule-mediated dynamic association between the Golgi complex and centrosome is of functional significance in cell polarization and division. Here, we summarize findings indicating how the Golgi complex and the centrosome cooperate in organizing the microtubule network for the directional protein transport and centrosome positioning required for cell polarization and regulating fundamental cell division processes.

## 1. Introduction

The Golgi complex (GC) is the central organelle of the secretory pathway and is involved in the processing and targeting of lipids and proteins [1]. In vertebrate cells, the GC consists of stacks of flattened cisternae that are laterally bridged by tubular “non-compact” zones that link equivalent cisternae [2] to form a single-copy organelle, the “Golgi ribbon”, which is often located adjacent to the centrosome and the nucleus [3].

Despite this complex organization, the GC can undergo many structural changes in response to specific physiological needs. For example, during cell migration, temporary Golgi unlinking is crucial when the organelle moves toward migration cues [4]. In addition, during the G2 phase of the cell cycle, the GC must be “unlinked” into isolated stacks to enable the onset of mitosis [5]. Then, during mitosis, the GC is disassembled into vesicular/tubular clusters that are dispersed in the cytoplasm until the so-called “Golgi haze” is formed; at the end of mitosis, the GC is reassembled to originate a new ribbon in each of the daughter cells [6].

The structure and location of the GC are controlled by the cytoskeleton. One of the first pieces of evidence of a functional connection between the GC and cytoskeleton is that the treatment with reagents able to depolymerize microtubules causes the redistribution of the perinuclear ribbon into dispersed ministacks [7]. Indeed, the microtubules control the localization and the organization of the GC in animal cells through several mechanisms. In most cell types, such as fibroblasts, the centrosome guides the formation of symmetric radial microtubule arrays [8]. Usually, the GC is maintained in a pericentrosomal location thanks to dynein-based centripetal transport along the radial microtubules formed by the centrosome [9]. At the same time, the GC can act as a microtubule-organizing centre to drive the polymerization of an asymmetric microtubule network [10]. While the radial microtubules mainly influence the Golgi ribbon positioning, the GC-originated microtubules act as tracks for the oriented delivery of post-Golgi cargo carriers [11], thus influencing cell migration and polarization and controlling GC organization [9]. Several studies demonstrated that if GC repositioning is inhibited, directed cell migration is blocked [12], supporting the idea that the plastic relocation of the GC is pivotal for many crucial cell functions.

During mitosis, the GC, the centrosome and microtubules, which are tightly interconnected, completely reorganize their redistribution. The centrosome and microtubules are the main actors of spindle formation, which is an indispensable track system for the correct inheritance of the Golgi proteins involved in ribbon formation. In addition, GC unlinking is required for entry into mitosis, and GC-associated proteins are required for correct spindle formation and proper cytokinesis [13]. Here, we review the crucial functional connection of the GC with the microtubule cytoskeleton and the centrosome, focusing on cell polarity and mitosis.

## 2. The Golgi Complex and Cytoskeleton: Structure and Functional Connection during Interphase

The maintenance of GC architecture requires the coordinated actions of Golgi-associated structural components and the cytoskeleton.

### 2.1. Structural Golgi Proteins

The structural components of GC are known as the “Golgi Matrix”; they include the Golgi Reassembly And Stacking Proteins (GRASPs) GRASP55 and GRASP65 and the golgins [14]. GRASP65 [15] and GRASP55 [16] are peripheral proteins containing an N-terminal myristoylated glycine crucial for GC association. GRASP65 localization at the *cis*-Golgi is mediated by the golgin GM130 [17], whereas GRASP55 is recruited to the *medial*/*trans*-Golgi by golgin-45 [18]. GRASP65 and GRASP55 are characterized by an N-terminal “GRASP domain”, formed by two PDZ domains and a C-terminal regulatory domain [19]. The first PDZ domain can homodimerize with the second PDZ domain of another GRASP protein, forming trans-oriented complexes that tether adjacent membranes [20]. This property can explain the proposed role of the GRASPs in the association of the cisternae into stacks [21] and the linking of the stacks into a ribbon [22], although the precise role of the GRASPs in stacks formation is debated [23]. The golgins are structural proteins with long coiled-coil domains that extend from the GC to tether cytoskeletal components and membranes [14]. When golgin functions are disrupted, the GC is disassembled [14,24]. However, golgins’ role in GC structure is probably indirect, as they are mainly involved in the tethering interaction necessary for membrane traffic [14]. As an example, the depletion of GM130 results in alterations of membrane traffic that indirectly cause ribbon unlinking [25].

A critical structural feature that impacts cell migration and mitosis entry is a dynamic equilibrium between ribbon formation and disassembly. The GRASPs drive the formation step, and this role can be inhibited by the phosphorylation of T222 and T225 of GRASP55 [26] and S274 of GRASP65 [27], favouring the unlinking step. In addition, the cleavage of the tubules connecting the Golgi stacks is actively controlled by the fission-inducing protein CtBP1-S/BARS [28,29]. The fine-tuning of the formation/cleavage equilibrium is crucial for the structural reorganization of the GC during cell migration, polarization and mitosis [30].

### 2.2. The Microtubule Cytoskeleton

**Figure 1:**
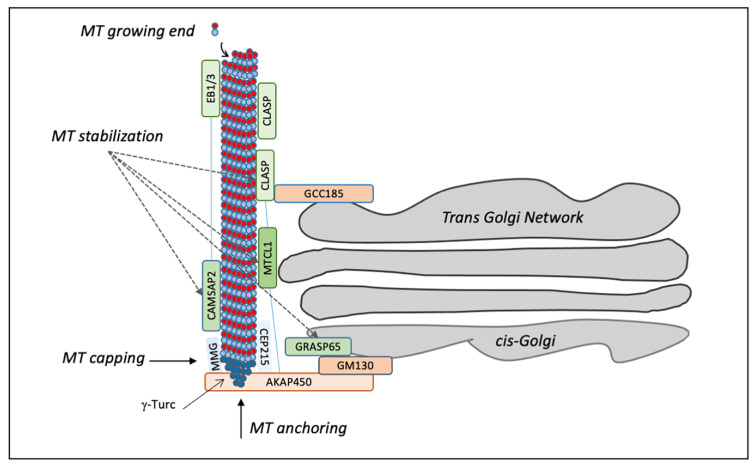
Schematic representation of microtubule polymerization at the Golgi. Microtubule fibres are polymerized starting from the γ-TuRC complex, which is responsible for capping the minus-end microtubules, while microtubules grow in the plus direction. GM130 at the *cis*-Golgi binds AKAP450, which in turn links CEP215 and MMG to recruit γ-TuRC for nucleation of Golgi-dependent microtubule. After their release from γ-TuRC, the microtubules are stabilized by CAMSAP2 and CLASP1/2, which are connected to the *trans*-Golgi through GCC185. The interaction between MTCL1 and CLASP1/2 can tether microtubules, whereas the length of the microtubules is regulated by CAMSAP2 when it associates with EBs proteins. The GM130 interactor GRASP65 regulates microtubule stabilization.

The microtubule cytoskeleton controls the localization and the organization of the GC in animal cells through several mechanisms [31]. Most of the relevant studies focused on fibroblasts, where the centrosomes form a radial microtubule network, which is responsible for the location of the GC near the centrosome. The centrosome is surrounded by pericentriolar material, composed of multiprotein complexes crucial for microtubule nucleation. They act as a structural hub to anchor nucleation templates, known as the “γ-tubulin ring complex” (γ-TuRC), which directs the assembly of tubulin dimers, acting as a cap of the minus-end microtubules, while microtubules growth continues in the plus direction (Figure 1) [32]. The process of microtubule nucleation is characterized by dynamic instability, which is a balance between the assembly and disassembly of the plus end of microtubules, controlled by a specific set of accessory proteins involved in the regulation of capping, polymerization and disassembly [33]. Centrosomal microtubules are crucial for the positioning of the GC, as they act as tracks for the centripetal movement of Golgi membranes, which is mediated by the minus end-directed motor complex dynein, recruited at the GC by golgin-160 [34] and AKAP450 [35]. In turn, the binding between golgin-160 and Arf1 is responsible for its association with Golgi membranes, and during mitosis, golgin-160 is dissociated from Golgi membranes, permitting their dispersal. Thus, the association of golgin-160 with the membrane through Arf1 is an essential control point during cell differentiation and polarization [34].

In addition, the GC can nucleate microtubules [36] through two protein complexes positioned at the *cis* and *trans*-sides of the stacks, respectively. A major protein complex is located at the *cis*-side by GM130, which recruits the scaffold protein AKAP450 to organize nucleation hot spots together with myomegalin (MMG) and CEP215 (also known as Cdk5rap2) (Figure 1). AKAP450 knockout cells or MMG depletion result in Golgi unlinking, traffic defects, and the accumulation of peri-Golgi vesicles [37,38]. CEP215 is better known for its localization at the centrosome [39], where it is involved in microtubule organization [40]. The newly nucleated microtubules are stabilized by a set of proteins, including CLASP1 and 2, which are microtubule plus-end tracking proteins associated with the *trans*-Golgi network by the golgin GCC185 (Figure 1) [41,42]. In addition, the GC-originated microtubules are stabilized by the microtubule-crosslinking protein (MTCL1) and the calmodulin-regulated spectrin-associated protein (CAMSAP), connected to the end-binding proteins (EBs) EB1, EB2 and EB3 (Figure 1) [43,44]. EBs are recruited at the GC also by MMG, which regulates the length of microtubules interacting with CAMSAP2 and inducing the spreading of GC along microtubules. MTCL1 knockdown reduces the number of microtubules around the nucleus, resulting in ribbon unlinking and polarization defects. MTCL1 interacts with CLASPs, AKAP450 and CAMSAP2, cooperating in microtubules stabilization [45]. Overall, these observations show the importance of the GM130–AKAP450–MMG–γ-TuRC complex in microtubules nucleation from the GC (Figure 1).

Notably, GRASP65, which interacts with GM130 and has a crucial role in ribbon formation [27], is also involved in stabilizing newly nucleated microtubules associated with the GC [46]. Stabilization leads to acetylation, which induces clustering of the Golgi stacks and favours ribbon formation. The ability of GRASP65 to induce ribbon formation and microtubules stabilization is regulated by ERK/JNK-mediated phosphorylation of S274 [46]. The depletion of GM130 induces the loss of microtubule acetylation, which is rescued by the expression of a short C-terminal domain of GM130 involved in recruiting GRASP65 at the GC [46].

There are also examples of additional, albeit less investigated, Golgi-based microtubule nucleation mechanisms. For example, in motor neurons, the ARF-mediated COPI-coat assembly induces the recruitment at the GC of tubulin-specific chaperone E (TBCE) to induce the nucleation of microtubules directed from the GC to the endoplasmic reticulum. TBCE mutations and the consequent alterations of tubulin assembly are associated with motor axon degeneration and motoneuron cell death, the causative defects of the Sanjad–Sakati/Kenny–Caffey syndrome, characterized by severe mental retardation and facial dysmorphism [47,48].

The Golgi-originated microtubules have two essential functions: (a) to induce the clustering of the stacks into a continuous ribbon [36,42]; and (b) to form fibres that are extended out of the GC and oriented toward specific plasma membrane domains [41]. This latter feature enables the cells to generate asymmetry for the polarized transport of post-Golgi carriers, which is fundamental for the correct function of particular cell types, such as neurons and muscle cells [49,50,51,52].

### 2.3. The Actin Cytoskeleton

Actin is involved in several processes, such as cell shape, migration, cytokinesis, endocytosis and exocytosis [53]. Several studies have demonstrated that the depolymerization of actin, using C2 botulinum toxin, cytochalasin D or latrunculin B, causes the swelling of the cisternae and compaction of the GC near the centrosome; on the contrary, when actin is stabilized, the cisternae are fragmented (for a broad review see [54]). The actin and actin-binding proteins are connected to the GC and its vesicles and perturbations of actin dynamics result in alterations of GC morphology [54]. During the last years, proteins regulating Golgi-associated actin have been found; for example, the Rho–mDia1 pathway [55], ADF/cofilin [56], and WASP homologue associated with actin, membranes and microtubules (WHAMM) [57]. The Rho–mDia1 pathway regulates the fusion of the Golgi membranes and the formation of Rab6-positive Golgi-derived transport carriers and is fundamental for GC integrity [55]. The activation and knockdown of the actin-depolymerizing factor ADF/cofilin induce variations in Golgi-mediated secretion and trafficking events [56].

The GC architecture and the transport from the endoplasmic reticulum to the GC are regulated by actin filaments nucleated by the Arp2/3 complex and its interactor WHAMM [57]. Furthermore, some GC-associated events are controlled by Golgi structural proteins that interact with actin and actin-associated proteins; for instance, GRASP65 interacts with actin elongation factor Mena to enhance local actin polymerization and Golgi ribbon linking [58]. Additionally, golgin GCC88 interacts with a scaffold protein intersectin-1 (ITSN-1) to regulate the dynamics of the GC structure [59].

Current evidence indicates that, at least in vertebrate cells, actin filaments control the GC morphology and rigidity [60]. Among the proteins that are important for the trafficking from GC to the plasma membrane, Golgi phosphoprotein 3 (GOLPH3) modulates GC organization by tuning the tensile forces exerted by actin on the Golgi membranes [59,61].

Taken together, these data suggest that actin at the GC controls both membrane traffic and ribbon morphology, even if the molecular mechanisms are only partially understood [62].

## 3. The Golgi Complex, the Centrosome and Microtubules Cooperate to Control Cell Polarity and Migration

In many cell types, the GC is located in the proximity of the centrosome. This arrangement is probably crucial for the directionality of membrane traffic from the GC [11]. For example, in most migrating cell types, the GC is positioned in the direction of migration with the centrosome in front of the nucleus [63]. In epithelial cells with apicobasal polarity, the GC is located between the nucleus and the apical surface, and specific traffic routes from the TGN to the plasma membrane are essential to maintain the polarity [11]. Additionally, during immunological synapse formation, the GC of a T-cell reorients toward the synapse formed with an antigen-presenting cell [64]. In pyramidal neurons, directed secretion from the GC to the axon is necessary to maintain the polarized organization of neuronal cells [11,49]. Instead, the GC is organized in isolated stacks with the *cis*-side facing the nuclear membrane in striated muscle cells, and colocalizes with the centrosome. However, in these cells the primary microtubule-organizing centre function is prevalently exerted by the nuclear envelope [65]. It has been suggested that, as the centrosome is pivotal to ensure the fidelity and proper orientation of cell division, it maintains a crucial role as a microtubule-organizing centre in the interphase of actively dividing cells. Conversely, in the differentiated cells that exit the cell cycle, several microtubule-mediated processes can be organized by centrosome-independent pathways, eliminating the need for a centrosomal microtubule-organizing centre [66].

**Figure 2:**
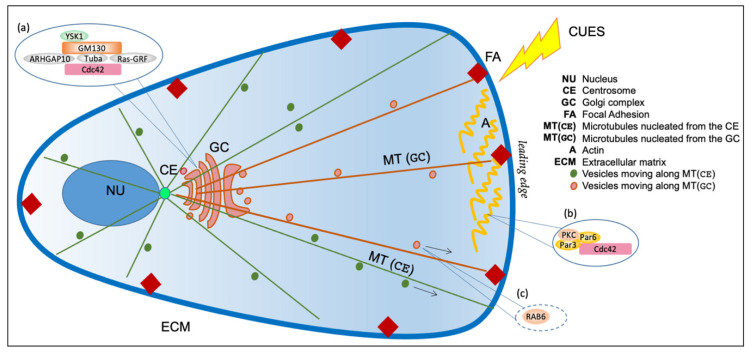
Schematic representation of Golgi complex with respect to the centrosome and nucleus. The Golgi complex (GC) is positioned near the centrosome (CE) in front of the nucleus (NU). The cytoskeleton comprises actin (A) and microtubules (MT). Actin controls membrane traffic and rigidity of the Golgi complex, while microtubules can be nucleated both from centrosome (CE) and Golgi (GC). The centrosome regulates the nucleation of a radial array of microtubules (green, MT (GC)) that maintain the pericentriolar localization of the Golgi Complex. The Golgi complex nucleates microtubules (brown, MT (GC)) asymmetrically oriented toward the leading edge. Insets (**a**,**b**) represent selected schematic depictions of protein complexes involved in polarized traffic localized at the Golgi complex (**a**) or at the plasma membrane (**b**); see the text for more details. Inset (**c**) shows a selected example of a crucial regulator of post-Golgi traffic, the small GTPases RAB6, which controls protein complexes involved directed secretion to hotspots closely associated with FAs, and that is also transported to FAs through MTs; see the text for more details.

Thus, as the role of the centrosome in differentiated non-dividing cells is less prominent, here we will mainly focus on the physical centrosome–GC relationship in the interphase of actively dividing cells. Many studies have shown that the intracellular organization and position of the GC and the centrosome are dictated by the extracellular matrix composition and stiffness and by polarization cues (Figure 2) [67].

In particular, the extracellular matrix geometry has a fundamental role in establishing the plasma membrane anisotropy, which is a crucial step to define the cell polarity. The anisotropy is a structural and molecular asymmetry at the cell surface obtained by extrinsic spatial cues, whose position defines a polarity axis along which the nucleus and centrosome are oriented, involving cell–cell and cell–extracellular matrix adhesion [68]. The spatial information of cues is propagated through the plasma membrane via focal adhesions (FAs), which are contact points between the cell membrane and the extracellular matrix (ECM), mediating the communication between the surrounding extracellular environment, the actin and microtubules cytoskeleton, thus modulating the global cell organization (Figure 2) [69].

The influence that the environment exerts on the geometry of the cell and the internal cell polarity and, in particular, the anisotropic distribution of cell adhesions, has been clearly shown by studies based on cells grown of fibronectin micropatterns, characterized by specific geometries and dimensions [70]. This approach demonstrated that specific micropatterns could modulate the cytoskeleton intracellular organization and organelles depending on the shape and the dimensions. For example, X-shaped micropatterns do not induce a specific symmetric axis and do not induce preferential orientation of the nucleus-centrosome–Golgi axis, resulting in a lack of polarization. Conversely, C- or K-shaped micropatterns are characterized by a single polarization axis, resulting in a defined and reproducible cell polarization [70]. These studies also showed that the asymmetrical patterns could result in an asymmetrical orientation of the spindle, generating unequal mitosis [71].

The extracellular matrix can influence cell organization not only based on its spatial organization, but also through the relative composition of glycosaminoglycans and fibrous proteins, which influence its mechanical rigidity, revealing a “rigidity mechanosensing” mechanism [72]. A related example is offered by a specific form of migration, called durotaxis, which is influenced by extracellular matrix–rigidity gradients [73]. Durotaxis is mediated by actin-based protrusions and a high turnover of focal adhesions that can sense the local environment and apply pulling force to guide the movement [74]. In turn, the membrane protrusions are also controlled by the persistent elongation of microtubules toward the leading edge, which also have several roles in regulating focal adhesion dynamics [75]. A recent study showed that in cells treated with Plk4 inhibitor centrinone-B [76], which is able to deplete the centrosome and thus ablate centrosome-dependent microtubules, the durotaxis process is no affected. On the contrary, Golgi-originated microtubules are required for the durotaxis, having the ability to modulate the focal adhesions turnover via post-Golgi trafficking toward the focal adhesion sites. Indeed, the depletion of the Golgi-originated microtubules induces an increase in focal adhesions area and hampers the turnover of focal adhesions [77].

A poorly understood aspect is how the GC is polarized toward the leading edge. During durotaxis, cells subjected to stiffness gradient exhibit coordinated Golgi–nucleus orientation toward the leading edge, supporting the idea that mechanical cues direct GC positioning in the migration process [78].

In addition to the geometrical and physical constraints exerted by the extracellular matrix, migrating cells can respond to a variety of stimuli, including wounding, electric fields or chemotactic gradients, so that their cytoskeleton and thereby their secretory system are reorganized to promote polarization and migration toward the cue [79]. Primary polarization cues, initiated at the cell leading edge, activate the GTPase Cdc42, triggering a Par6–Par3–PKC polarity complex [80]. This complex recruits and anchors dynein, which pulls on astral microtubules to reorient the centrosome and align the GC toward the leading edge (Figure 2) [81].

From the GC side, a key role is exerted by GM130, which recruits and activates the kinase YSK1, which phosphorylates downstream targets involved in cell polarity. The inactivation of YSK1 inhibits both GC and centrosome reorientation during cell migration (Table 1) [82]. GM130 also controls the organization of the centrosome in interphase through the Cdc42 exchange factors Tuba [83] and RasGRF [84]. Tuba, together with ARHGAP10, controls Cdc42 activation at the GC [85]. Similarly, RasGRF modulates Cdc42 activation at the GC, resulting in Ras–ERK activation [84]. Cdc42 localizes to the GC, where it is involved in protein transport and controls the centrosome reorientation during cell polarization (Figure 2) [86]. Depletion of GM130 reduces the activity of Cdc42 at the GC, but does not affect the plasma membrane pool of Cdc42 in the same cell [84]. These data underline the essential functional role of GM130, showing that its depletion causes the inhibition of directed motility, whereas, at the same time, it enhances random cell motility. Since the loss of cell polarity is associated with tumorigenesis and metastasis, it is noteworthy that impairment of the GM130–Cdc42 axis correlates with defects in cell motility (Table 1), which are particularly evident in breast and colon cancer [86].

Importantly, unlinking of the Golgi ribbon is required for GC reorientation. Indeed, during reorientation of the centrosome, GRASP65 is phosphorylated by ERK in S274, and the expression of a non-phosphorylatable form of GRASP65 blocks its reorientation [12]. Significantly, this block is neutralized if the GC is fragmented by brefeldin A, which induces artificial disruption of the GC [12]. The specific role of ribbon integrity and the GC association with the centrosome in cell migration have been often debated. Studies based on the expression of different domains of AKAP450, designed to impair ribbon formation or GC–centrosome association, suggested that the close linkage of GC with the centrosome, and not ribbon integrity, is the crucial factor for directed migration [87]. According to this view, the temporary GC unlinking during cell polarization would have a simple yet crucial facilitatory role.

These data corroborate the proposal of a strong functional connection among microtubules, the centrosome and extracellular matrix to assure the correct positioning of the GC in the direction of the cues so that the cargoes that are sorted at the *trans*-Golgi network can be delivered to specific plasma membrane domains. Indeed, secretion is required for maintaining cell polarity [89,90], and GC-originated cargoes are directed to the leading edge during the polarity response (Figure 2) [91]. The disruption of microtubules, which causes the fragmentation and dispersal of the GC, does not reduce the overall membrane traffic speed but affects cargoes targeting specific plasma membrane domains [92]. This concept has been directly shown by a recent study that demonstrated that RAB6-dependent post-Golgi cargoes, including VSV-G, NPY and TNFα, are driven along microtubules to secretion hotspots, close to focal adhesions. RAB6-dependent complex controls the secretion toward exocytic sites near focal adhesions, and its depletion affects the secretion process (Figure 2) [93].

The vital role of GC-originated microtubules is further supported by other studies involving the scaffold protein AKAP450 and the interactors CLASP1/2, CAMSAP2 and MTCL1, which cooperate to induce microtubule stabilization and acetylation [44]. The knockdown of MTCL1 causes a minor accumulation of perinuclear acetylated microtubules and disruption of the Golgi ribbon structure. This event induces unpolarized cell migration due to defects in polarized vesicle trafficking towards the leading edge (Table 1) [45]. As previously mentioned, other proteins that tether microtubules to GC are EB1, EB2 and EB3, which are associated with CAMSAP2 and influence the GC reorientation during cell polarization and migration [44]. The correct organization of microtubules at the GC is crucially regulated by the interaction of EBs with AKAP450 and MMG (Table 1) [38]. The depletion of the EBs impairs directional migration, and the knockout or abnormal expression of EBs induces an incorrect reorientation of GC, resulting in defects in cell polarity and migration [38,44].

## 4. The Golgi and Cytoskeleton: Structure and Functional Connection during Cell Division

One of the fundamental processes in cell proliferation and tissue growth is mitosis. Before starting this process, the cell must be reshaped from a flat to a spherical form during the G2/M transition [94]. The DNA duplication occurs before mitosis, during which period the DNA is equally redistributed into the daughter cells. The cytoskeleton, particularly the microtubules, is the major organizer of cell division. Specifically, microtubules are the main components of the spindle apparatus, which is involved in chromosome segregation [95]. Usually, the spindle microtubules are organized by the centrosomes, which are duplicated during the S-phase and separated at the two cell poles during G2. One of the main components of the pericentriolar material is pericentrin, which is necessary for spindle organization [96], together with additional core pericentriolar material components such as CEP192, CEP215, CEP152, SPD-5 and others [39]. The pericentriolar material is crucial for microtubule nucleation, acting as a structural hub to anchor the microtubule minus ends [97] and organizing three types of microtubule fibres: kinetochore, polar and astral microtubules [13]. Once assembled, the core pericentriolar material proteins can recruit additional components necessary for microtubule nucleation in a process called “centrosome maturation”.

The relative positioning of the GC and centrosome varies during the cell cycle [98]. Indeed, during G1, the GC is compact and localized near the centrosome; during S-phase, the GC dissociates from the centrosome, assuming a perinuclear and more extended localization, and surrounds the nucleus in early G2. Then, the GC membranes form two compact clusters that reassociate around each new centrosome in daughter cells at the G2/M transition. The different GC conformations, switching from compact to distributed morphology, are regulated by microtubules. It is likely that during G2 the centrosomes do not have an essential role in GC conformation transitions, as they are also observed in the absence of centrosomes [98].

**Figure 3:**
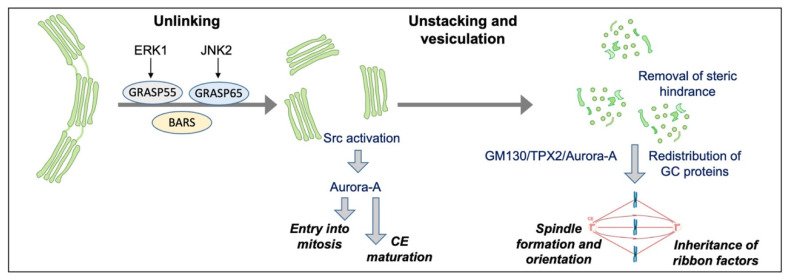
Schematic representation of GC mitotic partitioning and its functional connections with the regulation of mitosis. During the cell cycle, the GC undergoes sequential and reversible disassembly steps. During G2, the Golgi complex is unlinked process thanks to the action of BARS and the phosphorylation of GRASP65 by JNK2 and GRASP55 by ERK1. The G2-specific unlinking is required to activate a Src/Aurora-A mediated pathway to induce centrosome maturation and enter mitosis. After mitosis onset, the Golgi stacks are further disassembled into dispersed vesicles and clusters. Stack disassembly removes a steric hindrance that could hamper spindle formation; the GM130/TPX2/Aurora-A pathway is activated to aid in spindle formation and orientation. In addition, a set of Golgi-structural proteins relocates to the spindle and is repurposed for its proper formation. In turn, the spindle mediates the inheritance of ribbon factors for GC reformation at mitotic exit.

Recent studies showed that functional connections between the GC and centrosome influence specific cell cycle steps. For example, during G2, the Golgi ribbon must be separated into its constituent stacks to allow entry into mitosis, indicating that cell division is controlled by a “Golgi checkpoint”, which verifies correct Golgi ribbon separation in G2 [99]. The G2-specific ribbon separation activates a specific pool of the Src kinase, which in turn phosphorylates the residue Y148 of Aurora-A to induce its recruitment at the centrosome, promoting its maturation and triggering the activation of Cdk1, the primary regulator of mitosis (Figure 3) [100]. A crucial Aurora-A target involved in centrosome maturation is the scaffold protein CEP192, which recruits Aurora-A and Polo-like kinase 1 (Plk1), promoting the sequential activation of these kinases [101]. Local Plk1 activation results in the phosphorylation of CEP192 at several residues, inducing recruitment of the γ-tubulin ring complex and other pericentriolar material components, such as CEP215 [102]. Thus, a cooperative loop among Aurora-A, Plk1 and CEP192 promotes bipolar spindle formation [101], the geometry of which is controlled by the availability of active Aurora-A, which also integrates various stimuli to induce entry into mitosis [103]. When Aurora-A is depleted, the cells have supernumerary or defective centrosomes, showing aberrant mitotic spindle [104] and GC dispersal [105]. Of note, Aurora-A is an oncogene that promotes cell proliferation, acting on several substrates such as p53, Rb1, TPX2 and LAST2. Moreover, Aurora-A overexpression can reduce the efficiency of the DNA damage checkpoint through Plk1 activation and is involved in cell invasion and metastasis [106,107]. As Golgi unlinking induces Aurora-A activation regardless of the cell cycle phase [100], it would be essential to examine whether untimely or constitutive Golgi unlinking could contribute to cancer progression.

The functional GC–centrosome connection is not limited to the G2/M transition, but is also crucial after mitosis onset, when the Golgi stacks are further disassembled into dispersed vesicles and tubular/vesicular clusters. Indeed, the artificial block of GC unstacking after mitosis onset leads to the inhibition of centrosome separation, resulting in monopolar spindle formation and spindle assembly checkpoint activation, underlying the importance of GC disassembly after mitosis onset (Figure 3) [108].

While it is well known that the centrosome and the pericentriolar material have a fundamental role in microtubule nucleation in interphase, various studies indicated that a set of structural and Golgi-associated proteins during mitosis assume a direct role in the generation of spindle fibres (Figure 3) [13]. Among these proteins, a crucial role is attributed to the golgin GM130 and a set of binding partners, including p115, GRASP65 and AKAP450. GM130 has a direct role in spindle formation, thanks to a nuclear localization signal at the N-terminus. The Cdk1-induced phosphorylation of GM130 in Ser25 causes p115 dissociation from the nuclear localization signal. The dissociation of p115 is crucial for mitotic disassembly [109] and allows the nuclear localization signal to sequester importin-α at the GC [110]. This event liberates TPX2 from importin-α itself, allowing the interaction of TPX2 with Aurora-A and triggering spindle assembly (Figure 3) [51,110]. Furthermore, recent studies have demonstrated that GM130 is localized at the spindle and that blocking the GM130/importin-α/TPX2 pathway inhibits astral microtubule growth, causing spindle misorientation (Table 2) [111]. Thus, GM130 depletion results not only in centrosome reorientation defects during interphase [88], as previously mentioned, but also in spindle aberrations during mitosis (Table 2) [112]. The GM130-associated protein p115, mostly known for its role in GC structure and membrane traffic, dissociates from the Golgi membranes at mitosis onset and is portioned with the mitotic spindle. A peculiar feature of p115 is a short sequence, located at the N-terminus, involved in the binding to γ-tubulin and spindle targeting. Importantly, p115 depletion leads to GC disruption, in line with its prominent roles at the GC, but also in spindle defects, as a consequence of unstable spindle formation, and failed cytokinesis (Table 2) [113]. Among the interactors of GM130, also GRASP65 is involved in spindle organization and microtubule dynamics regulation, as GRASP65 depletion causes spindle alterations (Table 2) [114].

Recently, it was observed that GRASP65 has a central role in stabilizing Golgi-associated microtubules, permitting Golgi positioning and the formation of asymmetric microtubule fibres. In order to stabilize microtubules, GRASP65 requires a functional interaction with microtubule-binding proteins, such as CLASP2, although it is not clear if this interaction is direct. In agreement with this role, GRASP65 depletion results in a significant decrease in acetylated tubulin, a typical feature of stable microtubules. The correlation between GC and microtubules acetylation is crucial during the G2 phase; indeed, tubulin acetylation is strongly decreased during Golgi unlinking to help GC fragmentation [46]. Regarding AKAP450, this protein is crucial for astral microtubule dynamics and allows correct spindle orientation through the interaction with EB1 and p150; indeed, AKAP450 knockdown impairs the elongation of astral microtubules during anaphase (Table 2) [115].

As introduced before, CLASP1/2 are proteins that have a crucial role in microtubule plus-ends stabilization at the trans-Golgi network [116]. Importantly, their depletion results in spindle defects and cytokinesis failure [117], indicating that they also have essential roles in mitosis (Table 2). As reported above, WHAMM interacts with actin and microtubules, allowing membrane tubulation and active transport from ER to GC. A recent study demonstrated that the knockdown of WHAMM regulates spindle formation and influences the localization of the microtubule-organizing centre during the first steps of spindle formation. At the same time, WHAMM depletion causes spindle defects, resulting in aneuploidy and asymmetric division (Table 2) [118].

Altogether, these findings indicate that the functional interaction between GC and centrosome are crucial to drive correct spindle formation and assure the accurate segregation of the genetic material (Figure 3). In agreement with this hypothesis, a set of Golgi proteins must be released from the Golgi membranes to allow spindle formation and cytokinesis [119].

In turn, the centrosome and spindle have crucial roles in the inheritance of a set of Golgi membranes. Indeed, after ribbon separation, the cells enter mitosis, and the Golgi membranes are extensively disassembled into isolated vesicles and dispersed tubule–vesicular clusters [51,120]. While the enzyme-containing Golgi membranes are dispersed, probably in a microtubule-independent manner [121], a set of Golgi proteins essential for ribbon formation are recruited at the spindle, which mediates their inheritance [122]. Moreover, a specific sub-compartment of the secretory pathway, corresponding to the intermediate compartment, detaches during G2 from the bulk of the GC and associates with the centrosomes [123]. The precise role of this relocation is unknown, but it may play an important role in centrosome maturation by facilitating the Golgi–centrosome signalling. In addition, it has been recently suggested that the non-compact zones of GC could correspond to microtubule nucleation hotspots necessary for ribbon reformation at mitotic exit [124].

## 5. Concluding Remarks

The current evidence indicates that the close physical relationships among the GC membranes, the microtubules and the centrosome extend beyond a mere control of GC organization and has crucial roles for cell physiology. External cues influence the internal organization of the endomembrane–cytoskeleton system, including the position of the GC and the centrosome, to produce the proper directionality of protein secretion in interphase or the correct division axis in mitosis [125].

During interphase, the close association of the GC with the centrosome, mediated by the centrosome-originated microtubules, probably facilitates signalling efficiency between the GC and centrosome and thereby facilitates directional protein transport, which in turn requires asymmetrically directed and GC-originated microtubule tracks [11]. Thus, the GC–centrosome system may serve as a traffic hub, allowing the integrated regulation of exocytic and endocytic transport routes for microtubule-mediated polarized cargo delivery [126]. In support of this idea, in specialized cells where the centrosome loses its role, as in neurons, the GC is in the form of isolated stacks, “Golgi outposts” that can function as acentrosomal microtubule-organizing centres and nucleate a reticular microtubule network [11].

The importance of proper GC–centrosome–microtubule coordination is evidenced when its alteration results in the loss of polarized secretion. Thus, defects in Golgi redistribution and orientation may affect crucial physiological and pathological processes such as wound healing, embryonic development, axon determination, metastasis or immunological synapse formation [127].

An additional crucial GC–centrosome–microtubule functional relationship regulates mitosis. During G2, a Golgi checkpoint monitors the ribbon separation into stacks to allow entry into mitosis [5]. The essential signalling has been defined. Golgi unlinking induces the activation of a Golgi-localized pool of Src, which then phosphorylates the kinase Aurora-A to stimulate its recruitment at the centrosome to trigger entry into mitosis and centrosome maturation [100]. Then, after mitosis onset, the Golgi stacks undergo an extensive disassembly, which removes a steric hindrance that would impede correct centrosome separation. Indeed, blocking unstacking results in the accumulation of monopolar spindles and spindle checkpoint activation [108].

Despite the many unknowns of the GC-centrosome relationships, including how the GC-centrosome axis integrates the external signals to produce the necessary outputs, there are a few emerging general features. The first feature is that the GC-centrosome connection involves a bidirectional communication, as components of each organelle can influence the function of the other. For example, Golgi proteins are necessary for centrosome organization and positioning, and for mitotic spindle formation [13], whereas centrosome-nucleated microtubules are required for pericentriolar Golgi positioning [3].

**Figure 4:**
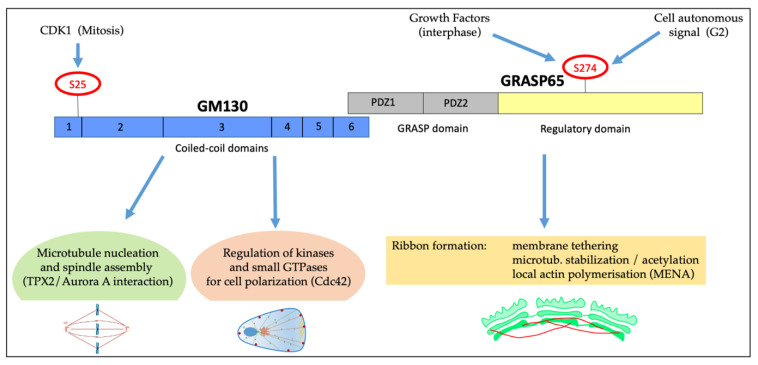
Schematic representation of GRASP65/GM130 complex and its role in polarization and mitosis. GM130 and GRASP65 form a complex at the GC and are involved in cell polarization and cell cycle progression. GM130, composed of six coiled-coil domains, is crucial for microtubules nucleation and spindle assembly and is involved in cell polarization. GRASP65, characterized by two PDZ domains (GRASP domain) and a regulatory domain, controls ribbon organization by multiple mechanisms: tethering of the Golgi stacks, control of microtubules stabilization and local actin polymerization via MENA. The S274 of the regulatory domain is phosphorylated in response to many stimuli, as indicated.

The second is the large variety of roles of the GM130/GRASP65 protein complex (Figure 4). During interphase, GM130 can activate the kinase YSK1 [82] and the exchange factors TUBA [83] and RasGFR [84] to regulate cell polarity, in coordination with the recruitment of AKAP450-based machinery to induce microtubule nucleation and control the relative GC–centrosome distance (Figure 4) [128]. In addition, GRASP65 supports ribbon formation through multiple mechanisms. For example, the ribbon unlinking is induced after the phosphorylation of Ser274 by various kinases, such as ERK after growth factors stimulation or stresses [12], or by JNK2 during G2 (Figure 4) [27]. At the same time, the GRASP65 regulatory domain binds the actin regulator Mena, which drives the formation of short actin fibres that facilitate Golgi linking [58]. The Mena-directed actin fibres preferentially operate on the short-range interaction of the stacks to maintain the integrity and modulate the “rigidity” of the GC [58]. Furthermore, GRASP65 induces microtubule stabilization, probably thanks to the interaction with microtubule-binding proteins, resulting in microtubule acetylation and clustering of the stacks (Figure 4). Notably, the microtubule-stabilizing role is inhibited by Ser274 phosphorylation [46]. In line with important roles, the depletion of either GM130 or GRASP65 results in massive spindle defects, indicating that these proteins are repurposed during mitosis to acquire novel roles in spindle formation [112,114]. While the GRASP65 role is unknown, GM130 binds the spindle, concurring to stabilize spindle fibres and activate a TPX2/Aurora-A pathway to control spindle orientation [111]. Thus, the GM130/GRASP65 protein complex has emerged as a critical hub to integrate a variety of signals and assemble protein complexes to output specific responses both in interphase and mitosis (Figure 4).

Although progress has been made, there are many unresolved questions about the Golgi–centrosome relationship. For example, how are GC-originated microtubules correctly oriented toward the proper plasma membrane domains? What is the initial signal that triggers G2-specific GC unlinking? What are the signalling pathways that regulate the centrosome–GC distance during the cell cycle, and how do they operate? Why is GC unlinking in G2 required for entry into mitosis? Is there a requirement for removing a steric hindrance, or is this necessary for the equal partitioning of GC–structural proteins? How accurate must the partitioning of GC proteins be? How are the roles of GM130 and GRASP65 coordinated during both interphase and mitosis? The answers to these questions will help us better understand the significance of the GC–centrosome–microtubule interactions and could lead to novel approaches for treating several important diseases, including cancer. Indeed, many pathological conditions can result from alterations of traffic routes deriving from Golgi–centrosome–microtubules axis unbalance, causing the mislocalization of proteins with crucial roles in cellular homeostasis. One example of this perturbation is observed in epithelial cell polarization, where a proper equilibrium among cell signalling, differentiation and trafficking is necessary for correct epithelial cell function.

## Figures and Tables

**Table 1:** Golgi-associated proteins involved in cell polarization and migration.

Protein	Depletion Phenotype	Reference
YSK1	Inhibition of GC and centrosome reorientation	[82]
GM130/Cdc42 axis	Defects in directed cell migration	[88]
MTCL1	Disruption of the Golgi ribbon and unpolarized cell migration	[45]
CAMSAP2	Reduced stability of Golgi-associated microtubules	[43]
golgin160	Reduced GC clustering and motility	[34]
MMG	Decreased directional migration	[38]
EB1, EB2, EB3	Defects in cell polarity and migration	[44]
AKAP450	Defects in cell polarity and migration	[87]

**Table 2:** Golgi-associated proteins involved in mitosis.

Protein	Depletion Phenotype	Reference
AKAP450	Impairment of astral microtubules in anaphase	[115]
GRASP65	Spindle alterations	[114]
CLASP1/2	Spindle defects and cytokinesis failure	[117]
WHAMM	Chromosomal aneuploidy and asymmetric division	[118]
GM130/Tuba/Cdc42 axis	Aberrant centrosome in interphase and defective spindle	[83]
GM130/importin-α/TPX2 axis	Inhibition of astral microtubule growth and spindle misorientation	[111]
p115	Unstable spindle formation and failed cytokinesis	[113]
